# Nutritional Value of Oregano-Based Products and Its Effect on Rabbit Performance and Health

**DOI:** 10.3390/ani14203021

**Published:** 2024-10-18

**Authors:** Ayman Abd El-Aziz, Ahmed Elfadadny, Mahmoud Abo Ghanima, Damiano Cavallini, Isa Fusaro, Melania Giammarco, Giovanni Buonaiuto, Karim El-Sabrout

**Affiliations:** 1 Department of Animal Wealth Development, Faculty of Veterinary Medicine, Damanhour University, Damanhour 22511, Egypt; ayman.sadaka@vetmed.dmu.edu.eg (A.A.E.-A.);; 2 Department of Animal Internal Medicine, Faculty of Veterinary Medicine, Damanhour University, Damanhour 22511, Egypt; ahmed.elfadadny@vetmed.dmu.edu.eg; 3 Department of Veterinary Medical Sciences, University of Bologna, Ozzano dell’Emilia, 40064 Bologna, Italy; 4 Department of Veterinary Medicine, University of Teramo, Piano d’Accio, 64100 Teramo, Italy; ifusaro@unite.it (I.F.);; 5 Department of Poultry Production, Faculty of Agriculture, Alexandria University, Alexandria 21545, Egypt

**Keywords:** carcass quality, essential oils, immunity, intestinal health, natural antioxidants, rabbit nutrition

## Abstract

**Simple Summary:**

Oregano essential oil has recently garnered attention for its potential health benefits in both humans and animals. In rabbits, it can significantly enhance performance by promoting overall health and well-being. The active compounds in oregano oil, such as carvacrol and thymol, possess antimicrobial, antioxidant, and anti-inflammatory properties, that support the immune system and protect rabbits from diseases. Additionally, oregano essential oil help regulate gut flora, promoting digestion and nutrient absorption, which enhances rabbit performance. However, proper dilution and dosage are crucial to ensure the safety and effectiveness of oregano essential oil in rabbits. With careful consideration and expert guidance, oregano essential oil can be a valuable natural remedy for supporting rabbit health and performance.

**Abstract:**

Antimicrobials long been used to enhance the performance and immunity of rabbits, typically by adding them to feed or drinking water to prevent illness. However, increasing consumer concerns about antibiotic-resistant microorganisms have led to a shift toward natural, eco-friendly, non-antibiotic feed supplements that can improve rabbit health and productivity. Recently, aromatic herbs and their extracts have gained considerable attention as natural antioxidants with growth-promoting and health-boosting properties. Essential oils and secondary metabolites play a central role in these effects, with *Origanum vulgare* (oregano) emerging as a standout option. Compared to synthetic alternatives, oregano is a natural, residue-free feed supplement with fewer harmful side effects. Its key bioactive components, thymol and carvacrol, have been shown to deliver significant benefits when incorporated into rabbit diets, particularly in improving production performance, immunity, and antioxidant capacity. Supplementing rabbit diets and drinking water with oregano essential oil (OEO) has been found to enhance growth performance, feed efficiency, and meat quality, while also lowering cholesterol levels and boosting antioxidant activity. Despite these promising results, research on the use of oregano in rabbit farming remains limited. This review aims to provide an up-to-date overview of the potential effect of oregano supplements, on growth parameters, carcass and meat quality, hemato-biochemical parameters, antioxidant capacity, immunity response, gut health, and gene expression in rabbits.

## 1. Introduction

Rabbit farming has become increasingly important as concerns about the sufficiency of global food supply and resources for meat production continue to grow. This industry plays an essential role in fulfilling the growing demand for meat as well as promoting efforts towards worldwide food security [1]. In countries like Italy and Egypt, rabbit production has expanded significantly due to the need to supply fresh, healthy meat while providing additional income for small-scale families and farms. However, the farming environment for rabbits exposes them to various stressors, that can compromise their well-being [2]. This stress is also associated with an increase in disease prevalence, leading to a greater reliance on medications, particularly antibiotics. Although some antibiotics have been used therapeutically to improve animal health and welfare, most have historically been administered for prophylactic purposes or to enhance growth rates and feed conversion efficiency. To ensure consumer safety and prevent the development of antibiotic resistance, several regions, such as the European Union, have banned the use of antibiotics as growth promoters in feed formulations [3]. Consequently, researchers have increasingly focused on investigating alternative feed additives for use in rabbit nutrition [4]. Currently, phytobiotics are being explored as a potential substitute for antibiotic growth promoters. Phytobiotics are defined as plant-based feed additives derived from herbs, spices, or other plant parts, such as leaves, roots, flowers, fruits, or their extracts, including essential oils. Over the years, various studies have demonstrated the potential of phytobiotic products as viable alternatives to antimicrobials in feed formulations [5,6,7,8]. The inclusion of these compounds in rabbit diets shows promise for improving various aspects of animal health, growth, and overall production efficiency [8]. Among the different types of phytobiotic additives examined, oregano (*Origanum vulgare*) has garnered the most research interest over time [9].

Despite the widespread use of oregano products, there is a lack of systematic summaries in the scientific literature on how this supplementation affects production performance and health. This review aims to address this gap by focusing on key areas such as productive performance; haemato-biochemical parameters and immune function indicators; carcass attributes and meat quality. Our review offers a comprehensive and up-to-date analysis of oregano-based products in rabbit nutrition, a topic that has not been thoroughly explored in previous studies. In contrast to the fragmented research currently available, we integrate findings from multiple sources to provide a holistic view of oregano’s influence on rabbit performance and health.

To gather the relevant literature, we conducted a systematic search of peer-reviewed journals, databases, and academic repositories using predefined keywords related to oregano supplementation, rabbit nutrition, and health parameters. Strict inclusion and exclusion criteria were applied to ensure that only high-quality, pertinent studies were included, establishing a robust foundation for our conclusions.

Through this systematic review, we seek to clarify the effects of oregano-based additives on rabbit nutrition and offer new insights and guidelines for developing targeted strategies aimed at enhancing rabbit performance, health, and welfare.

## 2. Oregano-Based Product Characterization

*Origanum* is a genus of herbaceous plants belonging to the mint family (*Lamiaceae*), native to the Mediterranean basin [10]. This genus includes several species that have been widely used since ancient times for culinary and medicinal purposes [11]. The most commonly recognized species is *O. vulgare*, known as oregano in most European countries. It is an aromatic perennial herb, 30–50 cm tall, with white or purple flowers, and opposite, lanceolate, toothed leaves [12].

Oregano is widely acknowledged as a medicinal herb due to its remarkable antimicrobial, antioxidant, hypocholesterolemic, and immune-boosting properties. The efficacy of oregano in treating a wide range of human diseases has been demonstrated in both in vitro and in vivo studies [13]. These beneficial effects are attributed to the active components found in the plant [9]. Oregano contains several bioactive compounds such as rosmarinic acid, linalool, thymol, carvacrol, tannins, flavonoids, triterpenes, and phenols, which together constitute over 80% of its essential oil content [13]. Numerous studies have reported the growth- and health-promoting effects of oregano in livestock, particularly in monogastric animals (e.g., pigs [14,15], chickens [16,17,18], and pets [19]), as well as in ruminants [20,21]. The principal components identified in oregano essential oil include geraniol, carvacrol, thymol, and β-caryophyllene, among others, with their average concentrations detailed in Table 1.

As shown in Table 1, carvacrol and thymol are the primary compounds, although their concentrations vary significantly depending on the subspecies used, cultivation methods, and the analytical methods of extractions [22,23,24,25,26,27]. These compounds possess notable antibacterial and antioxidant properties. Previous study [28] has documented the bactericidal characteristics of oregano essential oil, showing its ability to inhibit the growth of *Escherichia coli*, *Staphylococcus aureus*, and *Salmonella typhimurium*. Botsoglou et al. [16] found that including oregano essential oil in the diet decreased malondialdehyde levels in animal meat, indicating a protective effect against lipid oxidation. Furthermore, incorporating essential oils into livestock feeds may help reduce diarrhea in weaned animals and mitigate the severity of intestinal *Escherichia coli* infections [14]. The Panel on Additives and Products or Substances used in Animal Feed (FEEDAP) [24] concluded that the proposed use level of 35 mg/kg is safe for rabbits. No concerns regarding consumer safety were identified when the additive was used up to the maximum safe concentration in feed. Additionally, the use of essential oil extracted from *Origanum vulgare* ssp. in animal husbandry is not expected to pose a risk to the environment.

## 3. Beneficial Impacts of Oregano-Based Product Supplement

### 3.1. Productive Performance

Productive performance is a primary concern for farmers, particularly due to its direct impact on the profitability of rabbit farming. Optimizing growth rate and feed efficiency is essential for reducing feed conversion ratio (FCR) and improving farm profitability [29]. In intensive rabbit farming, variable costs such as feed, replacement stock, and artificial insemination account for more than 60–65% of total costs, with feed for growing rabbits being the most significant expense [29]. As a result, significant attention has been devoted to the use of feed additives in rabbit farming to enhance performance and reduce costs. Table 2 highlights several key studies that have investigated the effects of adding oregano-based products, including oregano oil, on productive performance.

Feed efficiency is a critical factor for enhancing both the economic and environmental sustainability of farming operations. In rabbit farming, feed costs are largely determined by the feed conversion ratio (FCR), which represents the ratio of feed intake to body weight gain. The FCR is a key metric for assessing the efficiency of converting feed into live weight gain, and it is widely used in farm-level performance evaluations as well as in nutrition experiments. Several studies (Table 2) have shown that the inclusion of oregano extracts in rabbit diets can significantly improve feed efficiency. For instance, Abdel-Wareth et al. [25] reported a reduction in FCR, from 3.60 in the control group to 3.12 in diets supplemented with 12 g/kg of dried oregano leaves or oregano bioactive lipid compounds (PBLC). Similarly, Omer et al. [30] evaluated the impact of adding oregano leaves to the diet of male New Zealand White rabbits, focusing on feed efficiency and diet digestibility. In their experiment, a basal diet was supplemented with 2% sunflower oil and 1% oregano leaves. While the dietary interventions did not significantly affect feed intake, crude protein, or ether extract digestibility, the rabbits fed oregano-supplemented diets demonstrated enhanced growth performance. Notably, the FCR improved from 3.59 in the control group to 2.84 in the oregano-supplemented group. According to the authors, this improvement in feed efficiency was attributed to either increased feed intake or enhanced body weight gain. Li et al. [31] also observed an increase in average daily feed intake with the inclusion of just 0.02% oregano essential oil (OEO). Moreover, Omer et al. [30] found that the final body weight of rabbits fed a diet with 1% dried oregano leaves was significantly higher (2509 g compared to 2287 g in the control group), with a greater body weight gain (1931 g vs. 1717 g in the control group). Cardinali et al. [31] also observed differences in live weight, with rabbits fed a standard diet reaching 2.27 kg, while those supplemented with 0.2% oregano extract achieved 2.34 kg. Similarly, Ayoub et al. [36] reported improved performance, particularly in terms of final body weight and average daily gain, compared to animals on control diets. Hekal et al. [34] investigated the effect of carvacrol—a monoterpenoid phenol predominantly found in oregano (see Table 1)—as a supplement in drinking water. The results from their study highlight the positive effects of this additive on both body weight and total feed intake in rabbits. According to the authors, rabbits supplemented with 0.26 mL carvacrol per liter of water showed a significantly higher weight gain (*p* ≤ 0.05) compared to the control group, while those supplemented with 0.13 and 0.39 mL carvacrol per liter exhibited the lowest weight gain [34]. All these results are particularly relevant for large-scale farming operations, where even modest improvements in feed efficiency can yield significant economic benefits. In addition to economic advantages, better feed efficiency results in more efficient resource use, contributing to the environmental sustainability of rabbit farming.

Finally, the study by Ayoub et al. [36] provides valuable insights into the addition of oregano essential oil in diets, particularly under conditions of stress such as high stocking density and mite infestation. The results demonstrated that the inclusion of oregano essential oil improved the condition of rabbits infested with mites, with those in the treatment group achieving a significantly higher final body weight (2.60 kg) compared to the control group (2.19 kg). Similar findings were reported by Łapiński et al. [37], who evaluated the effect of oregano extract in drinking water on the performance of rabbits infected with *Eimeria* sp.

### 3.2. Health Indicators

Hematological and biochemical parameters are essential and reliable indicators used to monitor and evaluate the health and nutritional status of animals. Table 3 summarizes key studies that have investigated the effects of oregano-based products, including oregano oil, on these hemato-biochemical parameters and immune function indicators.

According to Abdel-Wareth et al. [25], dietary supplementation with oregano-based products in the diets of growing rabbits led to a reduction in total cholesterol levels (72.48 mg/L with oregano leaves supplementation and 70.15 mg/L with PBLC supplementation), compared to the control group (86.57 mg/L). Ayoub et al. [36] also reported similar findings. Likewise, the blood concentrations of both GOT and GPT enzymes were reduced in the oregano-supplemented groups, indicating no hepatocyte damage. Additionally, the same authors observed a significant decrease in serum urea and creatinine concentrations compared to the control group, with the best results observed in rabbits supplemented with 9 g/kg of oregano leaves or the equivalent PBLC level of 138 mg/kg. These values, indicating liver and kidney function, initially increased before subsequently declining. Moreover, biochemical analyses of blood serum revealed no adverse effects on specific organs, such as the liver, kidneys, or heart, which could help explain the improved performance of the rabbits.

Omer et al. [30] similarly observed reduced blood cholesterol levels, corroborating the hypolipidemic effect of oregano essential oils, which may be due to the inhibition of hepatic 3-hydroxy-3-methylglutaryl coenzyme A (HMG-CoA) reductase activity—a mechanism already reported by other authors (e.g., Crowell [40]). However, Omer et al. [30] did not observe any other significant effects on blood parameters in rabbits. A particularly interesting hematological result was reported by Hekal et al. [34], who found that the addition of various doses of carvacrol (0.13, 0.26, and 0.39 mL/L) increased erythrocyte and leukocyte counts, as well as hemoglobin, hematocrit, and lymphocyte percentages. Rabbits consuming 0.39 mL carvacrol/L in their drinking water exhibited the highest counts of erythrocytes (6.27 × 10⁶/mm^3^), leukocytes (6.93 × 10^3^/mm^3^), hemoglobin (10.71 g/dL), hematocrit (36.95%), and lymphocytes (65.45%), followed by those drinking 0.26 and 0.13 mL carvacrol/L, respectively, compared to the control group. Overall, rabbits drinking 0.39 mL carvacrol/L recorded the best neutrophil/lymphocyte ratio (51.21), followed by those consuming 0.26 mL/L (52.24) and 0.13 mL/L (54.03), compared to the control group (56.99). The heterophil/lymphocyte ratio is widely recognized as a reliable indicator of stress in rabbits [41]. Heterophils play a key role in natural immunity and cellular defense against microbial infections, while lymphocytes are primarily responsible for antibody production and cytokine release. Similar findings were reported by Beghelli et al. [38], whose results suggest that the addition of oregano-based products may modulate the in-vitro immune response in rabbits.

In intensive rabbit production, intestinal issues caused by various stressors (e.g., environmental conditions, stocking density, or management practices) are among the leading causes of diarrhea and rabbit mortality [42]. To mitigate these health problems, farmers often resort to adding antibiotics to their feed. However, the widespread use of antibiotics has contributed to the development of antibiotic-resistant bacterial pathogens. Therefore, maintaining intestinal health in livestock without relying on antibiotics has become a significant challenge for researchers [21]. Li et al. [31] conducted experiments in which the dietary addition of 0.08% oregano essential oil significantly increased the villus-to-crypt (V/C) ratio in the ileum of meat rabbits, although no significant effects were observed in the V/C ratio of the duodenum and jejunum. Moreover, they found that oregano essential oil influenced the physical barrier of the jejunum by upregulating the expression of the JAM2 and JAM3 genes. Intestinal mucosal permeability, a key component of the intestinal physical barrier, is closely linked to the expression of tight junction proteins, including JAM proteins, which are part of the immunoglobulin superfamily. JAM proteins not only contribute to the structural integrity of tight junctions but also function as ligands for lymphocyte function-associated antigen-1 (LFA-1) [31]. The study also reported a significant increase in interleukin-2 (IL-2) levels, suggesting that oregano essential oil may enhance the immune function of the jejunum in rabbits. Similar findings were reported by Beghelli et al. [38] in a study involving 100 New Zealand rabbits, where improved intestinal and immune function was observed at 30 days of age. Li et al. [31] also observed an increase in the content of secretory immunoglobulin A (SIgA) in the jejunum and secretory immunoglobulin G (SIgG) in the ileum with the addition of oregano essential oil to rabbit feed. Both SIgA and SIgG play crucial regulatory roles in gastrointestinal mucosal immunity. Specifically, SIgA serves as a major immune effector at mucosal surfaces, providing a primary line of defense against pathogens. Meanwhile, SIgG promotes the phagocytosis of pathogens by immune cells and neutralizes bacterial toxins. These findings suggest that OEO may enhance the ability to neutralize pathogens and toxins, thereby improving anti-inflammatory and disease resistance mechanisms, promoting overall immune function, and supporting intestinal health.

In recent decades, the use of natural alternatives as feed additives to reduce parasite infestations in rabbits has gained significant attention [39]. Several studies have been conducted to evaluate the efficacy of these alternatives, yielding promising results (Table 3). For instance, Łapiński et al. [37] observed that adding oregano extract to drinking water led to a lower presence of coccidia (*Eimeria* sp.) in rabbit feces at 49 days of age. Specifically, at the final sampling (91 days), the lowest number of coccidia oocysts recorded was 210 oocysts/g of feces, compared to 308.6 oocysts/g in the control group. Moreover, post-mortem examinations of the liver and intestines revealed no pathological changes typically associated with coccidiosis. However, it is important to note that a more recent study by Lohkamp et al. [39] did not observe any anticoccidial effects of oregano oil as a feed additive for fattening rabbits when compared to a classical coccidiostat (Diclazuril) or a non-supplemented diet.

### 3.3. Carcass Attributes and Meat Quality

Several studies have demonstrated the effectiveness of oregano-based products in preserving and improving the quality of meat and meat products, primarily through their antioxidant properties [43]. Oxidative processes are a major cause of quality deterioration in meat, as they negatively affect flavor, color, and nutritional value, ultimately reducing shelf life [44]. Numerous studies have investigated the impact of adding oregano-based products on carcass and meat quality (Table 4).

Abdel-Wareth et al. [25] investigated the effects of different levels of oregano leaves and their plant-based bioactive lipid compounds (PBLC) on carcass traits and internal organs of male Californian rabbits at the end of the experimental period. The supplementation of oregano leaves and PBLC increased carcass yield (e.g., 657.06 g in the group fed 9 g/kg oregano leaves vs. 600.70 g in the control group). In contrast, the perirenal and scapular fat to body weight (BW) ratio was lower in the oregano-supplemented group than in the control. The liver-to-body weight ratio also decreased with higher levels of oregano leaves or PBLC compared to the control group. No significant differences were observed in other internal organs, such as the heart, spleen, kidneys, and head, between the treated and control groups. Similar findings regarding organ weights were reported by Aquino-López et al. [35], although these authors also noted differences in head weight. Regarding carcass yield, results consistent with those of Abdel-Wareth et al. [25] were observed by Cardinali et al. [32], who reported an increase in carcass yield with the addition of 0.2% oregano extract (60.8%) compared to the control diet (58.8%). Aquino-López et al. [35] also found increases, with carcass yield ranging from 44.72% in the control group to 50.66% with the addition of 20% oregano bagasse.

In terms of proximate composition, Cardinali et al. [32] reported differences in the composition of the Longissimus dorsi muscle, with an observed increase in moisture content and a decrease in protein content in the meat. However, no significant differences were found in the proximate composition, cholesterol, or mineral content of raw hind leg meat [32]. These findings align with those of Mattioli et al. [45], who also did not observe changes in the proximate composition of rabbit meat following the addition of 2% oregano extract. However, Mattioli et al. [45] noted that oxidative stability values were lower in the group supplemented with oregano extract (0.20 mg MDA/kg meat) compared to the control group (0.26 mg MDA/kg meat) in loin muscle. Interestingly, Cardinali et al. [32] also observed that oregano extract supplementation increased bone weight (41.3 g vs. 38.4 g in the control group), particularly in the femur (7.27 g vs. 6.71 g in the control group). Despite this increase in bone weight, the authors reported no significant differences in femur fracture resistance between the supplemented and control groups.

Regarding the technological characteristics of meat, Aquino-López et al. [35] reported that the addition of oregano altered the pH of meat at 24 h and 10 days post-mortem. The same authors also observed differences in the water retention capacity (WRC) of the meat. The antioxidant action of oregano on muscle fibers may have influenced the WRC, as it has been shown in species like chicken that the addition of antioxidants in the diet helps preserve membrane functionality, thereby enhancing their role as a semi-permeable barrier. However, these results are partially due to the fact that pH is directly related to WRC, which can vary according to the hydrolysis of proteins, leading to ammonia release, and the hydrolysis of lipids, resulting in fatty acid release. It has been suggested that essential oils can coagulate the cytoplasm by damaging lipids and proteins. Damage to the cell membrane may cause the release of macromolecules and lead to lysis, modifying the pH and directly impacting protein stability, which in turn affects WRC. Aquino-López et al. [35] also reported differences in meat color. In particular, they found that the meat of rabbits fed oregano essential oil and oregano bagasse had a more pronounced yellow index (b*) compared to the control group. However, several other authors [26,47] did not observe any differences in the meat color of rabbits fed oregano leaves or oregano essential oil in drinking water. This discrepancy may be due to the lower dose of oregano essential oil administered in these studies compared to Aquino-López et al. [35]. Additionally, the different varieties of oregano used, which may contain varying levels of phenolic compounds, could also explain the variation in meat color.

Soultos et al. [46] evaluated the effects of supplementing two levels (100 and 200 mg/kg) of oregano essential oil and found no substantial impact on performance indicators. However, oregano did affect certain sensory parameters of the meat. The authors reported that the control carcasses spoiled more rapidly than those from both oregano-supplemented groups. Initial signs of spoilage, characterized by the development of off-odors, were observed in the control group after the 6th day of storage (2.7 ± 0.48). The control samples were fully rejected on the 8th day of storage when the total viable counts (TVC) and yeast and mold counts exceeded 7 log CFU/cm^2^ and 6 log CFU/cm^2^, respectively. Interestingly, no oregano essential oil odor was detected in the meat of the dietary supplemented groups throughout storage. Oregano essential oil supplementation also positively affected slime formation on the external surface of rabbit carcasses. By day 8, all control group carcasses exhibited a visible slime layer (1.7 ± 0.46), whereas carcasses from rabbits fed 100 mg/kg of oregano essential oil had only begun to show slime formation (3.0). Carcasses from rabbits fed 200 mg/kg of oregano essential oil exhibited noticeably less slime formation, with slime becoming visible only on day 10 of storage (2.8). The authors also noted improved hygienic conditions in the meat, which were attributed to the antibacterial activity of oregano essential oil against both Gram-negative and Gram-positive bacteria. In fact, the microbiological results reported by Soultos et al. [46] highlighted that dietary supplementation with oregano essential oil strongly inhibited the growth of *Brochothrix thermosphacta*, with significant antibacterial activity against this microorganism evident even after 6 days of refrigerated storage. On day 8, when sensory rejection occurred, both experimental groups had significantly lower *Brochothrix thermosphacta* counts, approximately 1.5 and 3 log CFU/cm^2^ lower, respectively, than the control samples. Moreover, inhibition of *Pseudomonas* spp. growth was detectable only after 8 days of refrigerated storage, when *Pseudomonas* spp. counts on rabbit carcasses were approximately 1 log CFU/cm^2^ lower in the treated groups compared to the control. Similarly, LAB and *Enterobacteriaceae* counts in control samples were up to 1 log CFU/cm^2^ higher than in the meat of rabbits fed 200 mg/kg of oregano essential oil after 8 days of storage. The addition of 100 mg/kg of oregano essential oil in the rabbits’ diet also inhibited LAB and Enterobacteriaceae growth on the carcasses. However, throughout refrigerated storage, populations of *Brochothrix thermosphacta*, LAB, and Enterobacteriaceae in the meat of rabbits fed 200 mg/kg of oregano essential oil were consistently lower than in the meat of rabbits fed 100 mg/kg of oregano essential oil.

## 4. Highlights and Future Perspectives

**Figure 1:**
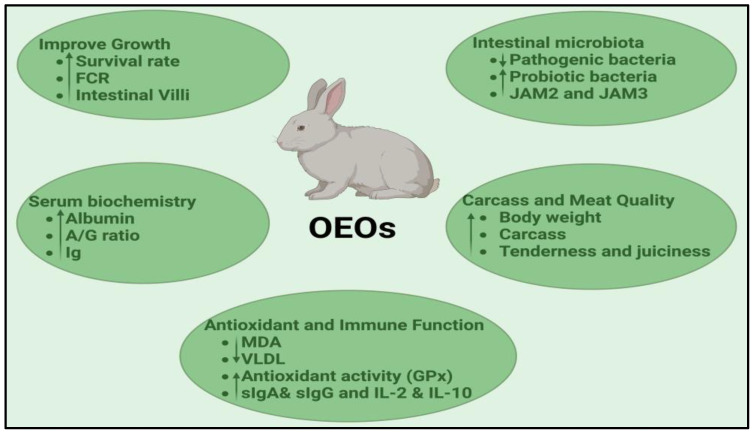
Beneficial effects of oregano essential oils (OEOs) for rabbit production.

As public concerns about antibiotic resistance and the use of synthetic additives in animal husbandry grow, attention has shifted towards natural, eco-friendly supplements, such as oregano products, which are likely to play an increasingly important role in modern agriculture. Oregano-based products present a promising natural alternative to synthetic antibiotics and growth promoters in rabbit farming (Figure 1). Studies mentioned in this paper demonstrated that incorporating oregano-based products into rabbit diets significantly improves key productive traits, including live weight, body weight gain, and feed conversion ratio. These findings suggest that oregano-based additives are effective in boosting productivity, which is crucial for reducing farming costs and improving profitability. Oregano’s ability to improve feed efficiency is especially valuable in intensive rabbit production, where feed expenses represent the primary cost. Moreover, oregano’s positive effects under stressful conditions, such as high stocking densities or mite infestations, further highlight its versatility as a growth promoter.

Additionally, oregano-based products exhibit strong antimicrobial, antioxidant, and anti-inflammatory properties [13], that contribute to improved immune function and overall health in rabbits. Oregano essential oil, for example, contains important bioactive fatty acids, such as linoleic, oleic, stearic, and palmitic acids. γ-Tocopherol constituted 32.1% of total measured tocols followed by α-tocotrienol (25.8%) and γ-tocotrienol (21.3%), as documented by [48]. More research is needed to fully understand the potential benefits of using these substances for rabbits and to determine the safe addition levels, either for parents or weaning/growing rabbits, and to prioritize the investigation of the fundamental mechanisms of action, refining supplementation techniques, and examining the potential synergistic benefits when combined with other dietary treatments. In addition, investigating the potential of additional bioactive chemicals found in oregano, such as carvacrol, might provide vital knowledge on innovative methods to improve the health and well-being of rabbits.

Research shows that oregano enhances antioxidant capacity, reducing oxidative stress markers in rabbit tissues [45]. Furthermore, oregano-based products improve blood profiles by lowering cholesterol, urea, and creatinine levels [25], indicating potential benefits for metabolic regulation and supporting liver and kidney function. The reduction in serum cholesterol is particularly noteworthy, as it suggests that oregano may act as a hypocholesterolemic agent, beneficial for both animal health and the quality of rabbit meat intended for human consumption. Oregano also exerts immunomodulatory effects, enhancing immune response indicators such as IL-2 and SIgA in the intestinal mucosa [31]. This supports gut barrier function and strengthens immune defense, making rabbits more resilient to infections and intestinal diseases. This effect is particularly important for reducing the reliance on antibiotics for disease prevention, aligning with global efforts to combat antibiotic resistance. Oregano’s positive impact on gut health is well-documented; it promotes the growth of beneficial gut bacteria while inhibiting harmful pathogens like *Escherichia coli* and *Salmonella typhimurium* [28]. This improved balance of intestinal microbiota leads to enhanced nutrient absorption and reduced incidence of diarrhea, a common issue in intensive rabbit farming. Notably, oregano shows promise in the prevention of coccidiosis [37], a parasitic infection affecting rabbit intestines that often requires treatment with synthetic anticoccidials. While some studies have reported limited efficacy of oregano against coccidiosis compared to conventional treatments, others highlight its potential as part of an integrated parasite control strategy, suggesting that oregano could be combined with other treatments to reduce parasite burdens and improve overall health, as demonstrated in this current article.

The antioxidant properties of oregano-based products also contribute to improved meat quality by preserving flavor, color, and nutritional value [35]. It reduces oxidative processes in meat, leading to rancidity and spoilage and positively influences carcass traits such as yield and composition. Rabbits supplemented with oregano-based products tend to have higher carcass weights and better meat quality characteristics, including improved water retention and color stability. These traits are crucial for maintaining meat quality during storage and extending shelf life, making oregano an attractive additive for meat producers.

Despite these promising results, further research is needed to fully understand the mechanisms behind oregano’s effects on rabbit performance, health, and meat quality. Future studies should focus on elucidating the molecular pathways through which oregano’s bioactive compounds exert antimicrobial, antioxidant, and anti-inflammatory effects. Additionally, investigating potential synergistic interactions between oregano and other feed additives could also provide valuable insights for optimizing rabbit nutrition. Another area of interest is the impact of oregano on reproductive performance and the health of breeding rabbits. While most studies focus on growing rabbits, understanding how oregano supplementation affects breeding stock, and their offspring could help develop new strategies for enhancing the sustainability and efficiency of rabbit farming.

According to the European Food Safety Authority (EFSA) opinion [24], a proposed use level of 35 mg/kg of essential oil extracted from *Origanum vulgare* ssp. *hirtum* in complete feed is considered safe for rabbits. However, more research is required to determine the optimal dosage and delivery methods for oregano-based products. While current studies provide valuable data on inclusion levels in feed and water, further work is needed to refine these recommendations to maximize efficacy and safety. For instance, exploring the long-term effects of oregano supplementation on rabbit health and performance could help establish guidelines for continuous use in commercial farming systems.

Furthermore, investigating the potential of combining oregano with other natural alternatives, such as probiotics or prebiotics, could open new avenues for improving gut health and disease resistance. Developing novel oregano-based formulations that incorporate additional bioactive compounds may also lead to more effective and targeted strategies for enhancing rabbit performance and welfare. However, the literature on the use of oregano-based additives reveals considerable variability in experimental protocols and their reported effects on animal performance and metabolism. Standardizing these protocols will be critical for advancing research in this area.

## 5. Conclusions

This study aimed to summarize scientific evidence from peer-reviewed journals on the use of oregano-based supplements in rabbits and their ability to enhance performance, support immune function, and improve meat quality. Oregano essential oil supplementation, particularly, has a positive impact on the digestive system of rabbits. It also shows promising potential, as a natural green alternative to antibiotics, for promoting health and immunity in rabbits. Oregano has been shown to possess anti-inflammatory properties and can help improve nutrient absorption in the gut. Research has indicated that oregano contains powerful antioxidants and antimicrobial properties, which can help support the immune system and protect against infections. By incorporating oregano into the diet of rabbits (leaves ≤ 0.5%; oil ≤ 75 mg/kg) or drinking water (oil ≤ 0.5 mL/L), it may be possible to enhance their overall well-being and potentially reduce the risk of certain health issues. Oregano supplementation also has the potential to boost disease resistance and productivity in rabbit farming by increasing immune responses and intestinal barrier integrity. Taken together, including oregano supplementation in rabbit nutrition shows potential for sustainable and comprehensive approaches, in line with the increasing need for natural alternatives and the advancement of animal welfare in agriculture. However, the evidence regarding oregano supplementation for rabbits is promising, continued research is essential to fully understand the mechanisms behind oregano’s beneficial effects and to optimize its use in commercial farming systems.

## Figures and Tables

**Table 1:** Principal components identified in oregano-based additives, according to scientific literature.

Compound (%)	Sarikurkcu et al. [22]	Llana-Ruiz-Cabello et al. [23]	EFSA [24]	Abdel-Wareth et al. [25]		Rotolo et al. [26]	
				Leaves	PBLC ^1^	Essential Oil DH ^2^	Essential Oil OD ^3^
1-Octen-3-ol	0.43	-	-	-	-	0.35	-
Borneol	1.01	-	-	0.17	0.41	2.78	0.98
Camphene	0.14	-	-	0.19	0.36	0.02	0.03
Carvacrol	16.11	55.82	78.1	85.1	53.3	23.5	23.4
Caryophyllene oxide	0.4	-	-	0.08	0.39	-	-
Linalool	-	-	3.41	-	-	-	-
p-Cymen	13.45	16.31	6.11	0.06	-	-	-
p-Cymen-8-ol	0.2	-	-	-	-	-	-
Terpinen-4-ol	-	1.33	0.67	0.09	0.28	3.12	7.38
Thymol	58.31	5.14	2.63	0.13	0.16	-	-
α-Pinene	0.23	1.1	0.8	0.12	0.27	0.23	0.24
α-Terpinene	1.13	1.62	0.6	0.62	2.72	0.89	2.68
α-Thujene	0.38	1.69	-	0.84	1.04	2.45	2.62
β-Bisabolene	0.21	-	-	0.08	0.42	2.61	3.86
β-Caryophyllene	2.22	2.4	1.67	0.49	1.96	1.50	2.44
β-Myrcene	-	1.52	0.93	0.34	2.94	0.98	0.58
γ-Terpinene	4.64	4.71	3.73	3.19	14.41	4.92	7.08

^1^ PBLC: Plant bioactive lipid compounds; ^2^ DH: Essential oil extracted from dehumidifying process; ^3^ OD: Essential oil extracted from oven-drying process.

**Table 2:** Key studies on the effects of oregano-based feed and water additives on productive traits.

Studied Traits ^1^	Product and Inclusion Level	Reference
LW and FCR	Oregano leaves: 3, 6, 9 or 12 g/kg PBLC ^2^: 46, 92, 138 or 184 mg/kg	Abdel-Wareth et al. [25]
GP	Oregano and sage dried leaves: 1%	Rotolo et al. [26]
GP, FCR and diet digestibility	Oregano leaves: 1%	Omer et al. [30]
ADFI	Oregano essential oil: 0.08%	Li et al. [31]
LW and CW	Oregano extract: 0.2%	Cardinali et al. [32]
BWG	Oregano extract: 1 or 3 mL	Garcia et al. [33]
GP and FCR	Carvacrol: 0.13, 0.26, or 0.39 mL/L of water	Hekal et al. [34]
LW and FCR	Oregano essential oil: 0.25 or 0.40 g/kg Oregano bagasse: 20%	Aquino-López et al. [35]
GP and stress resilience	Oregano essential oil: 200 mg/kg	Ayoub et al. [36]
BWG and meat quality	Oregano powder concentrate: 0.5 g/L of water	Łapiński et al. [37]

^1^ LW: Live weight; FCR: feed conversion ratio; CW: Carcass weight; GP: Growth performance; ADFI: Average daily feed intake; BWG: Body weight gain; BW: Body weight. ^2^ PBLC: Plant bioactive lipid compounds.

**Table 3:** Key studies on the effects of oregano-based feed and water additives on haemato-biochemical parameters, and immune function indicators.

Studied Traits ^1^	Product and Inclusion Level	Reference
Blood cholesterol, GOT, GPT, Urea, Creatinine	Oregano leaves: 3, 6, 9 or 12 g/kg PBLC ^2^: 46, 92, 138 or 184 mg/kg	Abdel-Waret et al. [25]
Blood cholesterol and LDL	Oregano leaves: 1%	Omer et al. [30]
V/C value, IL-2, SIgA in jejunum, IL-10 and SIgG in ileum.	Oregano essential oil: 0.08%	Li et al. [31]
Erythrocytes, Hemoglobin; Hematocrit, Leukocytes, Lymphocytes and N/L ratio	Carvacrol: 0.13, 0.26, or 0.39 mL/L of water	Hekal et al. [34]
Blood cholesterol, Mite infestation	Oregano essential oil: 200 mg/kg	Ayoub et al. [36]
Coccidia infection	Oregano powder concentrate: 0.5 g/L of water	Łapiński et al. [37]
Increase in immune response indicators	Oregano extract: 0.2%	Beghelli et al. [38]
IL-2	Oregano extract: 0.2%	Beghelli et al. [38]
Coccidia infection	Oregano oil: 75 mg/kg	Lohkamp et al. [39]

^1^ GOT: Glutamic oxaloacetic transaminase; GPT: Glutamic pyruvic transaminase; LDL: Low density lipoprotein; V/C: villus-to-crypt; N/L ratio: Neutrophil/Lymphocyte ratio; IL-2: Interleukin-2; SIgA: Secretory immunoglobulin A; IL-10: interleukin-10; SIgG: Secretory immunoglobulin G; ^2^ PBLC: Plant bioactive lipid compounds.

**Table 4:** Key studies on the effects of oregano-based feed and water additives on carcass and meat quality.

Studied Traits ^1^	Dosage	Reference
CY	Oregano leaves: 3, 6, 9 or 12 g/kg PBLC ^2^: 46, 92, 138 or 184 mg/kg	Abdel-Waret et al. [25]
CW, CY, Proximate composition	Oregano extract: 0.2%	Cardinali et al. [32]
CW, CY, pH, WRC, color	Oregano essential oil: 0.25 or 0.40 g/kg Oregano bagasse: 20%	Aquino-López et al. [35]
TBARs	Oregano extract: 2%	Mattioli et al. [45]
Sensory properties	Oregano essential oil: 10 or 200 mg/kg	Soultos et al. [46]

^1^ CY: Carcass yield; CW: Carcass weight; WRC: Water retention capacity; TBARs: Oxidative stability. ^2^ PBLC: Plant bioactive lipid compounds.

## Data Availability

The data can be made available from the corresponding author upon reasonable request.
